# The Cytotoxic Assessment of Antibacterial-Enhanced Mineral Trioxide Aggregate Compared to Commercially Available Bioceramic Cements by Using Methyl-Thiazoldiphenyl-Tetrazolium (MTT) Assay on Human Dental Pulp Stem Cells: An In Vitro Study

**DOI:** 10.7759/cureus.49691

**Published:** 2023-11-30

**Authors:** Ayushma Chakravorty, Vignesh Ravindran, Ganesh Jeevanandan, Abirami Arthanari

**Affiliations:** 1 Department of Pediatric and Preventive Dentistry, Saveetha Dental College and Hospitals, Saveetha Institute of Medical and Technical Sciences, Saveetha University, Chennai, IND; 2 Department of Forensic Odontology, Saveetha Dental College and Hospitals, Saveetha Institute of Medical and Technical Sciences, Saveetha University, Chennai, IND

**Keywords:** mineral trioxide aggregate, biodentine, diseases, dental, tricalcium silicate, metronidazole, doxycycline

## Abstract

Background and objective

Preserving the vitality of the tooth is of prime significance during therapies such as direct pulp capping and pulpotomy that promote tertiary dentine formation and healing of pulp stumps. Procedures like apexogenesis and apexification also stimulate dentin and bone formation for root growth and closure. Conventional mineral trioxide aggregate (MTA) has good biocompatible and physical properties like longer setting time, presence of a cytotoxic component, i.e., tricalcium aluminate (TCA), moderate compressive strength, and moderate antimicrobial activity. Eliminating TCA and the addition of antibacterial components would improve the properties of the cement. In this study, we aimed to assess the cytotoxicity of MTA Angelus, Biodentine, and two antibacterial-enhanced MTAs by using methyl-thiazoldiphenyl-tetrazolium (MTT) assay.

Materials and methods

Human dental pulp was extirpated from extracted third molars, and human dental pulp stem cells (HDPSCs) were isolated and characterized by flow cytometry. HDPSCs were treated with MTA, Biodentine, or two antibacterial-enhanced MTAs depending on the study group. The control group constituted the untreated HDPSCs. The cell viability of HDPSCs was assessed using an MTT assay on days one, three, and seven.

Results

Varied levels of cytotoxicity were noticed at different time periods assessed using the tested materials, which was statistically significant (p=0.01). At all time periods assessed, the highest cell viability was noticed with Biodentine (88.7% on the first day, 80.4% on the third day, and 91.8% on the seventh day). Antibacterial-enhanced MTAs, either added with metronidazole or doxycycline, had more mean viable cells compared to conventional MTA on the third and seventh day (p=0.043 and 0.018 respectively).

Conclusion

Antibacterial-enhanced MTAs showed reduced cytotoxic properties when compared to conventional MTA. Biodentine was associated with the highest cell viability at all time periods.

## Introduction

The fields of clinical medicine and dentistry have long struggled with the therapeutic regeneration of human cells, tissues, and dental pulp [1]. The ability to self-renew and the pluripotent potential, or capacity to differentiate and define into endothelial cells, odontoblasts, osteoblasts, etc., that can be used for regenerative purposes, were discovered in stem cells derived from teeth [2]. Dental pulp, periodontal pockets, and apical papilla are potential sources of these dental stem cells [3]. Early in the new millennium, dental pulp stem cells were isolated [4], which provided researchers the impetus to investigate other study possibilities.

Direct pulp capping and pulpotomy requiring reparative dentine formation for the healing of pulp stumps would be necessary for preserving tooth vitality. Apexogenesis and apexification also would require the formation of dentin and bone for apical closure [5]. human dental pulp stem cells (HDPSCs), which can support the course and outcome of the aforementioned therapy techniques, are commonly available from pulp and apical papilla [6]. The ideal requirement of such capping materials must include the provision of a hermetic seal and encouragement for HDPSCs to differentiate and proliferate to initiate healing processes [7]. Pulpal inflammation due to carious lesions or traumatic injury can induce healing and regeneration processes only when maintained under mild to moderate limits [8]. Materials like conventional mineral trioxide aggregate (MTA), Biodentine, ProRoot MTA, MTA Plus, MTA Angelus, and TheraCal have been used to maintain inflammatory responses, which have shown mixed clinical results [9].

Chemicals like chlorhexidine gluconate, tetracycline, nitric oxide-releasing compounds, calcium hydroxide, and recently the incorporation of fluorohydroxyapatite and calcium fluoride have been utilized to improve antimicrobial properties [10-12]. Metronidazole, a broad-spectrum class nitroimidazole, can be lethal against gram-positive anaerobes like E. faecalis, a common endodontic pathogen that is responsible for endodontic reinfections [13]. Suggestions on combining metronidazole with glass ionomer cements and antibiotic intracanal medicaments have been implemented, providing diverse results [13,14]. Another antimicrobial, doxycycline is a hydroxyl derivative of tetracycline, used as an adjuvant in irrigants and intracanal medicaments like triple antibiotic paste over the past decade [15]. Only one study has been identified related to this, which suggests that doxycycline did not potentially increase the antimicrobial activity of the final setting of Biodentine cement [16]. According to previously published research, augmenting antimicrobial properties has compromised physical strength [17]. This may be due to the vehicle used for drug delivery, which commonly involves the replacement of the liquid component by a commercially available product or powdered version of the commercially available drug [17].

Cytotoxicity assays, involving both in vitro and in vivo methods, are commonly used for studying material toxicity, cell viability, and tissue irritations [18]. The methyl-thiazoldiphenyl-tetrazolium (MTT) assay works on the basis of the mitochondrial activity of live cells which translates to the number of viable cells measuring the cytotoxic effect of the drug in an in vitro setup [19]. In vitro cytotoxicity tests are considered to be more reliable for evaluation as they are simple to perform, cost-effective, and easily reproducible [9].

Conventional MTAs have good biocompatible and physical properties like longer setting time [20], presence of a cytotoxic component, i.e., tricalcium aluminate (TCA) [21], moderate compressive strength, and moderate antimicrobial activity. Its drawbacks were recently addressed by the authors with the elimination of TCA and the addition of other agents, which showed promising results [22]. Enhancing these cements with the addition of antimicrobial agents during the manufacturing processes has not been attempted, and it would require cytotoxic assessment to justify clinical usage. In light of this, this study aimed to assess the cytotoxicity of MTA Angelus, Biodentine, and two antibacterial-enhanced MTAs by using the MTT assay.

## Materials and methods

Experimental design

Human dental pulp was extirpated from extracted third molars and HDPSCs were isolated and characterized by flow cytometry. HDPSCs were treated with either conventional MTA, Biodentine, or two antibacterial-enhanced MTA combinations depending on the study group. Untreated HDPSCs served as the control group. MTT assay was performed based on manufacturer instructions to assess the percentage of viable HDPSCs that were treated by the test materials (Abcam, Inc, Cambridge, UK) to test the effect of the test materials used in the present study on the cell viability of HDPSCs. This assessment was done at one-day, three-day, and seven-day incubation periods in the MTT reagent. This in vitro study design was approved by the members of the institutional ethical committee (approval number: SRB/SDC/PhD/Pedo/2022/045).

Materials tested

Two commercially available bioactive bioceramics and two newly formulated modified MTAs were used in the present study.

Commercially Available Materials Used in the Present Study

Group 1: MTA was obtained from Angelus (Angelus Indústria de Produtos Odontológicos, Londrina-PR, Brazil). It is packed as a powder-liquid formulation. As recommended by the manufacturer, a 3:1 powder-to-liquid ratio was dispensed on a pad. The powder was completely hydrated by the liquid until the mix turned to be thick, similar to a putty-like consistency. The completely mixed material was then carried using an MTA carrier to the desired experimental design.

Group 2: Biodentine was obtained from Septodont (Saint Maur des Fossés, France). It is also available as a powder-liquid packaging. As recommended by the manufacturer, five drops of the liquid were poured into the capsule containing the powder and mixed using a mechanical triturator (Dentsply Maillefer) for roughly 30 seconds. Once the mix is complete, it will have a thick consistency similar to putty. The material was then carried using a plastic instrument to the desired experimental design. 

Antibacterial-Enhanced MTAs Used in the Present Study

Group 3: Doxycycline-incorporated MTA formulation - the newly formulated composition of MTA includes tricalcium silicate, dicalcium silicate, calcium carbonate, calcium sulfate, and calcium fluoride as the base powder components. The core components, i.e., tricalcium silicate and dicalcium silicate, were manufactured in the lab based on the manufacturing process suggested by Moon et al. [21]. Doxycycline and calcium chloride were procured in powder form by TCI Chemicals India Pvt. Ltd. Doxycycline was added to 1 ml of distilled water separately to obtain a 5% concentration. Calcium chloride was mixed with 1 ml of distilled water to obtain a 20% concentration. Both the liquids were mixed until a uniform mixture was obtained. The composition is summarized in Table 1. Based on trial and error ratios, 100 mg of the proposed powder content and 40 µl of the liquid component were dispensed on a pad for the mix. After the complete hydration of the powder with the liquid, the mixing procedure was continued until a uniform mix with a moldable consistency was obtained. The material was then carried using a plastic instrument to the desired experimental design.

**Table 1:** Composition of doxycycline-incorporated MTA formulation used in the current study (group 3) MTA: mineral trioxide aggregate

Group 3	
Powder	Weight % for every 100 mg of powder
Tricalcium silicate	60 wt %
Dicalcium silicate	20 wt %
Calcium fluoride	5 wt %
Calcium sulfate	5 wt %
Calcium carbonate	4 wt %
Zirconium oxide	1 wt %
Liquid	Concentration
Calcium chloride	20%
Doxycycline	5%

Group 4: Metronidazole-incorporated MTA formulation - the powder component was similar to the composition provided in group 3. Metronidazole was procured in powder form from TCI Chemicals India Pvt. Ltd. The obtained Metronidazole was added to 1 ml of distilled water separately to obtain a 1% concentration. Calcium chloride was mixed with 1 ml of distilled water to obtain a 20% concentration. Both the liquids were mixed until a uniform mixture was obtained. The composition is summarized in Table 2. Based on trial and error ratios, 100 mg of the proposed powder content and 40 µl of the liquid component were dispensed on a pad for the mix. After the complete hydration of the powder with the liquid, the mixing procedure was continued until a uniform mix with a moldable consistency was obtained. The material was then carried using a plastic instrument to the desired experimental design.

**Table 2:** Composition of metronidazole-incorporated MTA formulation used in the current study (group 4) MTA: mineral trioxide aggregate

Group 4	
Powder	Weight % for every 100 mg of powder
Tricalcium silicate	60 wt %
Dicalcium silicate	20 wt %
Calcium fluoride	5 wt %
Calcium sulfate	5 wt %
Calcium carbonate	4 wt %
Zirconium oxide	1 wt %
Liquid	Concentration
Calcium chloride	20%
Metronidazole	1%

All the mixed materials were placed at the bottom of 6-well tissue culture plates, and dried under laminar flow for 48 hours at room temperature. 

Group 5: The control group, which contained untreated cells that followed a regular apoptotic cycle.

Cell culture

Freshly extracted human adult third molars were collected from the outpatient department of a private dental institute. A signed written informed consent was obtained from the patients regarding the use of the extracted tooth for research purposes, which was also approved by the institutional ethical committee. The teeth were sectioned at the cementoenamel junction for removal of pulp tissue from the exposed pulp chamber. The extirpated pulp tissue was then digested in a solution of 4 mg/ml dispase (Sigma-Aldrich, Burlington, MA) and 3 mg/ml collagenase type I for a duration of one hour at 37 °C. The cells were grown in α-MEM growth medium (UFC Biotech, Riyadh, KSA) that contained a 10% concentration of fetal bovine serum and 100 U/ml penicillin, 100 μg/ml streptomycin, and incubated at 37 °C in 5% carbon dioxide. This was followed by characterization using flow cytometry, similar to the study performed previously [7], to confirm that the isolated cells were positive for mesenchymal stem cell lineage.

Flow cytometric surface marker expression analysis in HDPSCs

HDPSCs that were cultured in the medium were analyzed for cell surface antigen expression, i.e., CD90 and CD45. Fluorescein isothiocyanate-conjugated mouse anti-human CD90 and phycoerythrin-conjugated mouse anti-human CD45 (BD Biosciences, Franklin Lakes, NJ) were utilized. HDPSCs were detached from the plate by using 0.25% trypsin with 1 mM EDTA (Gibco, Thermo Fisher Scientific, Waltham, MA), and 50 μl of cell suspension was mixed with 5 μl of the corresponding antibody. After incubation for 30 minutes in the dark, the cells were washed and then acquired by flow cytometry (CYTOMICS FC 500 Flow Cytometer, Beckman Coulter, Brea, CA) and analyzed using Cyflogic software version 1.2.1

Cell viability assay

MTT assay (Abcam) was performed based on manufacturer instructions to test the effect of the test materials used in the present study. MTT penetrates viable cells and reduces to form formazan. The amount of formazan dye formed is directly proportional to the number of metabolically active cells [23]. At 70% confluency, HDPSCs were suspended in α-MEM growth medium and seeded at 5 × 104 cells/well, and incubated with the test materials in 24-well plates containing a final volume of 900 μl/well. Untreated cells were used as the control group. The cells were incubated at 37 °C in 5% carbon dioxide for 24 hours. At 24 hours, 100 μl MTT stock solution (5 mg/ml) was added to attached HDPSCs in each well to achieve a final concentration of 0.5 mg/ml and incubated for three hours at 37 °C. At the end of the incubation period, the medium was removed, and dimethyl sulfoxide:isopropanol [24] solvent solution was added to dissolve formazan crystals. The solution was transferred to a 96-well plate at 100 μl/well and optical density was read at 570 nm by a spectrophotometric Microplate Reader (SpectroStar Nano, BMG Labtech, Ortenberg, Germany). The same methodology was repeated after a three-day and seven-day incubation period.

Statistical analysis

The percentages of viable HDPSCs in the test group materials to untreated control were assessed using one-way analysis of variance (ANOVA) in the IBM SPSS Statistics software version 22 (IBM Corp., Armonk, NY). The experiments were done in triplicate and the results were expressed as mean and standard deviation (SD). A p-value <0.05 was considered statistically significant.

## Results

**Figure 1:**
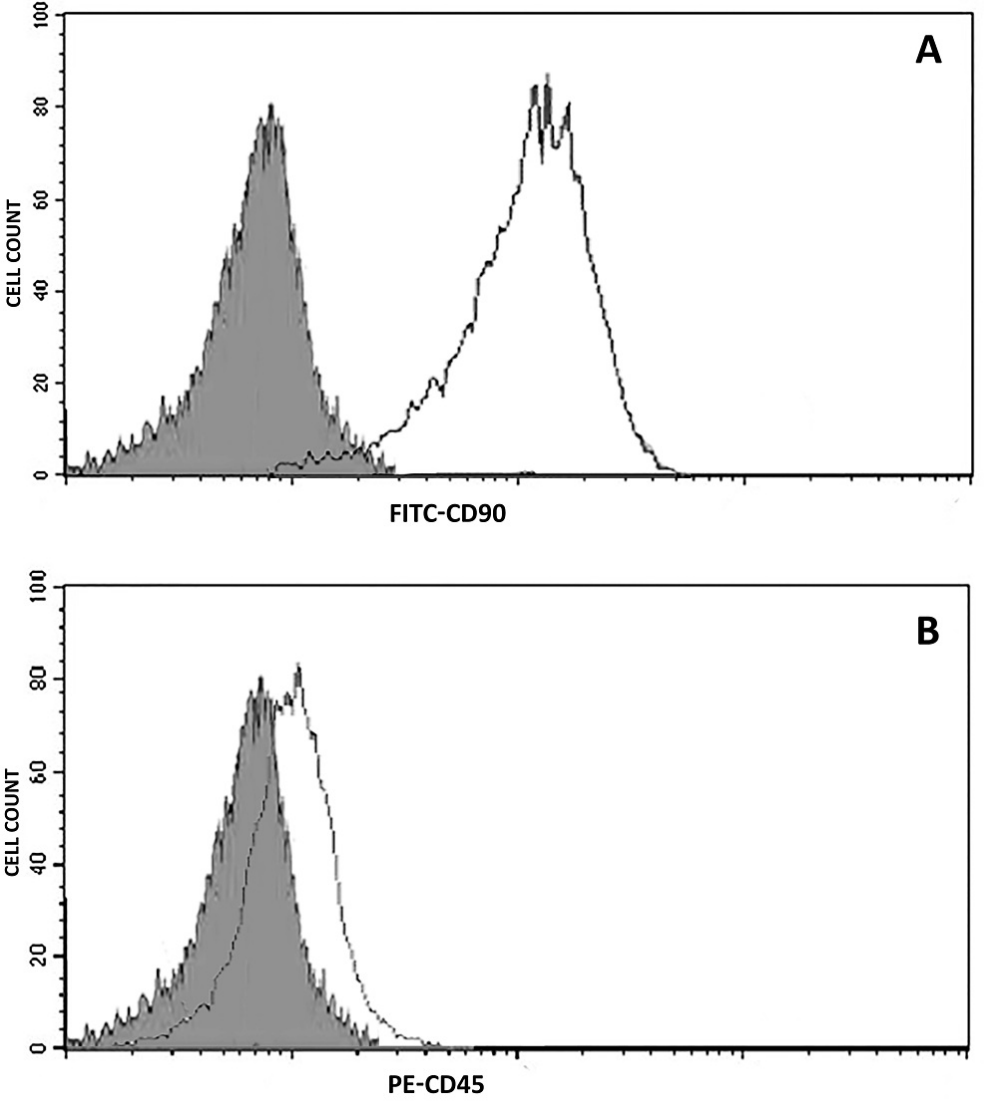
Flow cytometry histograms of expression of HDPSCs for mesenchymal lineage (A) and hematopoietic lineage (B) Filled histograms represent unstained control cells while the empty histograms represent the cells stained with antibodies against surface protein CD90-FITC and CD45-PE in A and B respectively. There is a positive peak shift for the marker CD90 for mesenchymal lineage and a negative for the marker CD45 for hematopoietic lineage HDPSCs: human dental pulp stem cells

HDPSCs that were cultured in α-MEM growth medium were strongly positive for mesenchymal stem cells (MSC) marker CD90 to confirm the mesenchymal lineage, which were only included for testing in the present study (Figure 1).

**Figure 2:**
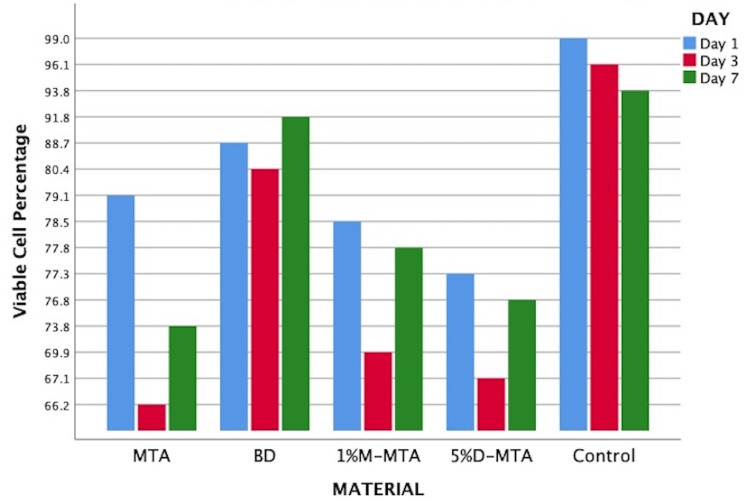
Graph representing the cell viability percentage among the materials tested on days 1, 3, and 7 MTA: mineral trioxide aggregate; BD: Biodentine; 1%M-MTA: 1% metronidazole-enhanced MTA; 5%D-MTA: 5% doxycycline-enhanced MTA

Compared to the very high percentages of viable cells in cell control, varied levels of cytotoxicity were noticed at the different time periods assessed using the tested materials. There was a uniform reduction in the cell viability in the control group during the three time periods due to the regular apoptotic cell death. During the first day of assessment, there was a considerable reduction of viable cells in all the materials tested. This reduction of viable cells increased by the end of the third day in all the test groups except the Biodentine group, which had only a minimal reduction of viable cells. Also, this reduction by the third day was higher for the conventional MTA as compared to the antibacterial-enhanced MTA. However, a substantial regeneration of cells was noticed by the end of the seventh day in all the test groups. This regeneration of HDPSCs was higher for the antibacterial-enhanced MTA as compared to the conventional MTA. This was found to be statistically significant overall (p=0.01) (Figure 2).

Intragroup comparison, i.e., comparison within the materials at different time periods, showed considerable reduction in viable cells by the end of the first day and third day while an appreciable regeneration was noticed by the end of the seventh day, which had no statistical significance. Despite the lack of statistical significance in intragroup comparisons, intergroup comparisons showed significant differences. Among the test materials, at all time periods assessed, the highest cell viability was noticed with the Biodentine group (88.7% on the first day, 80.4% on the third day, and 91.8% on the seventh day). By the end of the first day, MTAs enhanced with 5% doxycycline and 1% metronidazole had similar reductions in mean viable cells (77.3% and 78.5% respectively) as compared to conventional MTA (79.1%). This comparison was not statistically significant (p=0.112). By the end of the third day, MTAs enhanced with 5% doxycycline and 1% metronidazole had more mean viable cells (67.1% and 69.9% respectively) as compared to conventional MTA (66.2%). This comparison was found to be statistically significant (p=0.043). By the end of the seventh day, MTAs enhanced with 5% doxycycline and 1% metronidazole had significantly more mean viable cells (76.8% and 77.8% respectively) as compared to conventional MTA (73.8%). This comparison was found to be statistically significant (p=0.018) (Table 3).

**Table 3:** Evaluation of cell viability among the materials tested in the present study at different time periods MTA: mineral trioxide aggregate

Group	Material	Cell viability (%)			P-value
		1st day	3rd day	7th day	
Group 1	MTA (Angelus, Londrina-PR, Brazil)	79.1	66.2	73.8	0.54
Group 2	Biodentine (Septodont, Saint Maur des Fossés, France)	88.7	80.4	91.8	0.24
Group 3	5% doxycycline-enhanced MTA	77.3	67.1	76.8	0.34
Group 4	1% metronidazole-enhanced MTA	78.5	69.9	77.8	0.16
Group 5	Control	99.0	96.1	93.8	0.91
	P-value	0.112	0.043	0.018	

## Discussion

Due to its widespread use in contemporary clinical practice, the assessment of cytotoxic and biocompatibility properties of bioactive cements has been in trials in recent decades. HDPSCs, due to their clonogenic and pluripotent activities, have played an important role in tissue healing. The material’s biocompatible properties are the key factors determining the viability of such HDPSCs that are required for tertiary dentin formation [25,26]. Such HDPSCs were isolated and tested in the present study. Based on ISO standards, cell viability tests can be evaluated at the end of 24, 48, and 72 hours [27]. In the present study, we assessed till the end of the seventh day to evaluate the long-term cytotoxic effects on HDPSCs of the materials that were tested.

Antibiotics have long been used in dental practice for disinfection in the form of irrigants, intracanal medicaments, and also in addition to other dental cements [14]. Chlorhexidine has been attempted before to be combined with dental cements but did not lead to any improvement of the final cement in terms of physical properties and cytotoxicity [17]. E. faecalis, being the most resistant endodontic pathogen to many antibiotics used, has been shown to be lethal to nitroimidazole like metronidazole [13]. Tetracycline groups of drugs, like minocycline and doxycycline, have long been used as a component in the combination of triple antibiotic pastes [15]. This was the rationale for using metronidazole and doxycycline in the present study, which showed better results compared to conventional MTA.

Several studies in the literature have periodically assessed the biocompatibility of such MTA-based cements due to their high necessity in our daily endodontic-related dental procedures [25,28,29]. These studies show that MTT assay remains the preferred in vitro methodology to assess the cytotoxic effects of MTA-based bioceramic cements in the past decade. The MTT assay is an in vitro assessment of the total mitochondrial activity of the cells, which interprets the number of viable cells for the measurement of cytotoxicity of the materials tested [19]. It is easy to perform, reproduce, and cost-effective [9,30].

In the current study, Biodentine showed the least cytotoxicity at various time periods assessed. Antibacterial-enhanced MTAs (addition of either metronidazole or doxycycline) were found to be less cytotoxic than conventional MTA. The reduced cytotoxicity could be attributed to the elimination of TCA, which was concluded in an earlier study [21]. As this is the first of its kind to assess the cytotoxicity of antibacterial-enhanced MTAs, we were unable to find other similar studies for comparison of our results. However, studies using different bioactive cements have been performed, which show varied results [25,28,29]. Rodrigues et al. have compared MTA Plus and MTA Angelus on HDPSCs using an MTT assay and concluded that both materials did not have significant cytotoxic effects [28]. They also suggested that although both materials had a slight increase in necrotic cells, they did not induce apoptosis [28]. Jung et al. compared MTA, BioAggregate, and Biodentine using an MTT assay and concluded that all the materials had a similar effect on cell viability [29]. Chang et al. evaluated BioAggregate, Micromega MTA, ProRoot MTA, and intermediate restorative material by MTT assay for a 14-day period and concluded that all the test materials had similar biocompatibility except for intermediate restorative material [25].

The current study assessed the toxicity properties of antibacterial-enhanced MTAs on HDPSCs, which are essential for tissue healing during pulp therapies. The present study has a few limitations, primarily its in vitro study design. We recommend that animal studies be conducted to assess the regenerative properties before initializing human trials. Further studies on the correlation of chemical components of the cements and their biological responses toward cells are required.

## Conclusions

Antibacterial-enhanced MTAs showed reduced cytotoxic properties when compared to conventional MTA. Biodentine was associated with the highest cell viability at all time periods. Enhancing the properties of such bioactive cements can be hugely beneficial and will lead to clinically superior results, and further studies are required in this regard.
